# Metabolic responses and “omics” technologies for elucidating the effects of heat stress in dairy cows

**DOI:** 10.1007/s00484-016-1283-z

**Published:** 2016-11-30

**Authors:** Li Min, Shengguo Zhao, He Tian, Xu Zhou, Yangdong Zhang, Songli Li, Hongjian Yang, Nan Zheng, Jiaqi Wang

**Affiliations:** 1 State Key Laboratory of Animal Nutrition, Institute of Animal Science, Chinese Academy of Agricultural Sciences, Beijing, 100193 People’s Republic of China; 2 State Key Laboratory of Animal Nutrition, College of Animal Science and Technology, China Agricultural University, Beijing, 100193 People’s Republic of China

**Keywords:** Heat stress, Dairy cows, Body metabolism, Endocrine profiles, Metabolomics, Proteomics

## Abstract

Heat stress (HS) negatively affects various industries that rely on animal husbandry, particularly the dairy industry. A better understanding of metabolic responses in HS dairy cows is necessary to elucidate the physiological mechanisms of HS and offer a new perspective for future research. In this paper, we review the current knowledge of responses of body metabolism (lipid, carbohydrate, and protein), endocrine profiles, and bovine mammary epithelial cells during HS. Furthermore, we summarize the metabolomics and proteomics data that have revealed the metabolite profiles and differentially expressed proteins that are a feature of HS in dairy cows. Analysis of metabolic changes and “omics” data demonstrated that HS is characterized by reduced lipolysis, increased glycolysis, and catabolism of amino acids in dairy cows. Here, analysis of the impairment of immune function during HS and of the inflammation that arises after long-term HS might suggest new strategies to ameliorate the effects of HS in dairy production.

## Introduction

**Fig. 1:**
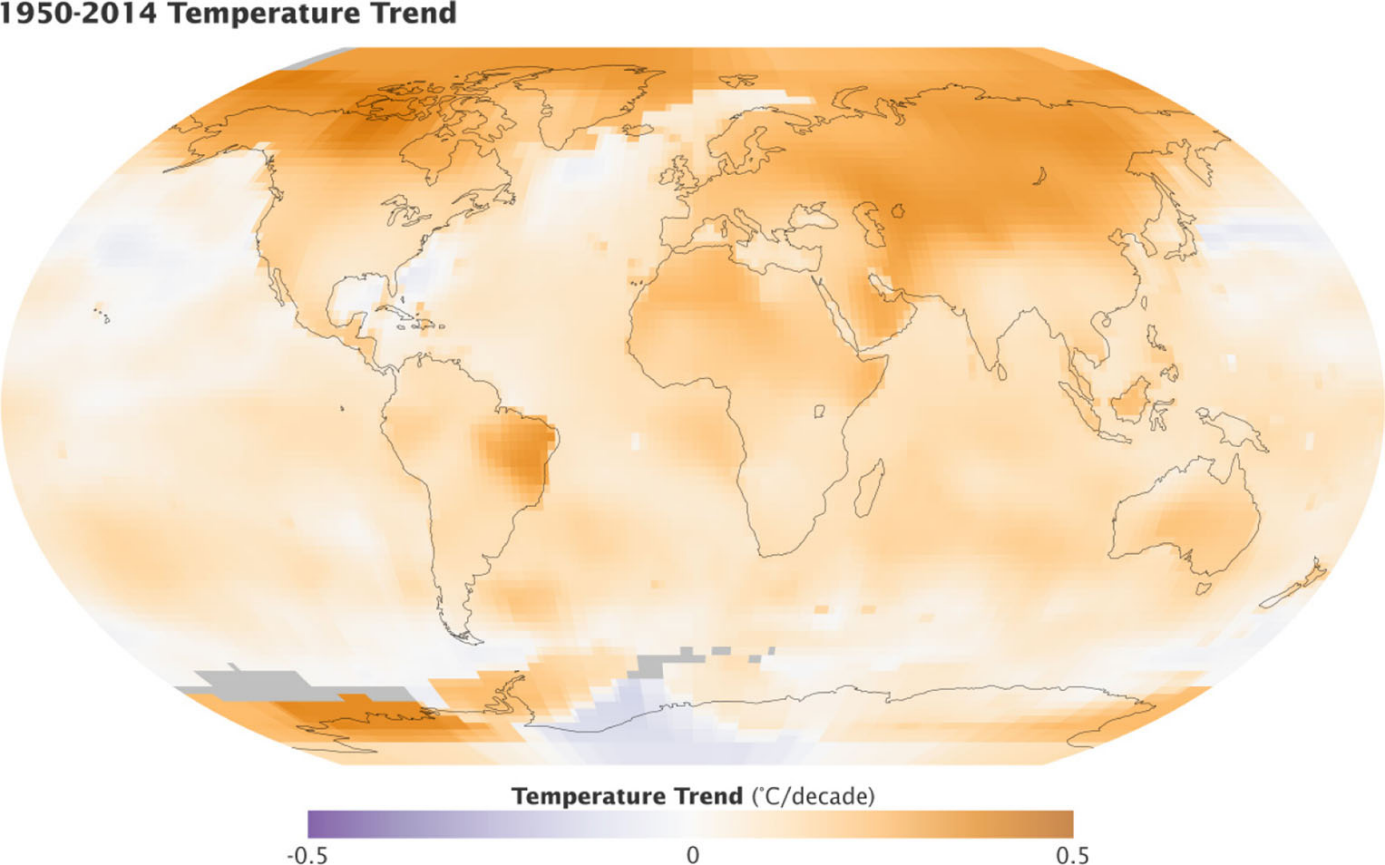
World map showing surface temperature trends (°C per decade) between 1950 and 2014 (derived from the National Aeronautics and Space Administration) (NASA 2015). Global temperature rose at an average rate of about 0.13 °C per decade in the last 50 years

Multiple lines of scientific evidence indicate that the global climate is warming (Hartmann et al. 2013; Sherwood and Huber 2010; Dunne et al. 2013). The world map in Fig. 1 shows trends in surface temperature (°C per decade) between 1950 and 2014 (derived from the National Aeronautics and Space Administration) (NASA 2015). The global temperature rose at an average rate of approximately 0.13 °C per decade over the last 50 years. In 2014, the combined land and ocean surface temperature was 0.69 °C above the twentieth century average, making the year the warmest since records began in 1880. In addition, scientists project that the global average temperature will rise by around 0.2 °C per decade over the next 20 years (Dahlman 2009). Summer temperatures have been increasing worldwide, and this trend is predicted to continue (Luber and McGeehin 2008). According to the results of modeling by the Intergovernmental Panel on Climate Change, heat stress (HS) is projected to increase in terms of both severity and number of incidents (IPCC 2007).

**Fig. 2:**
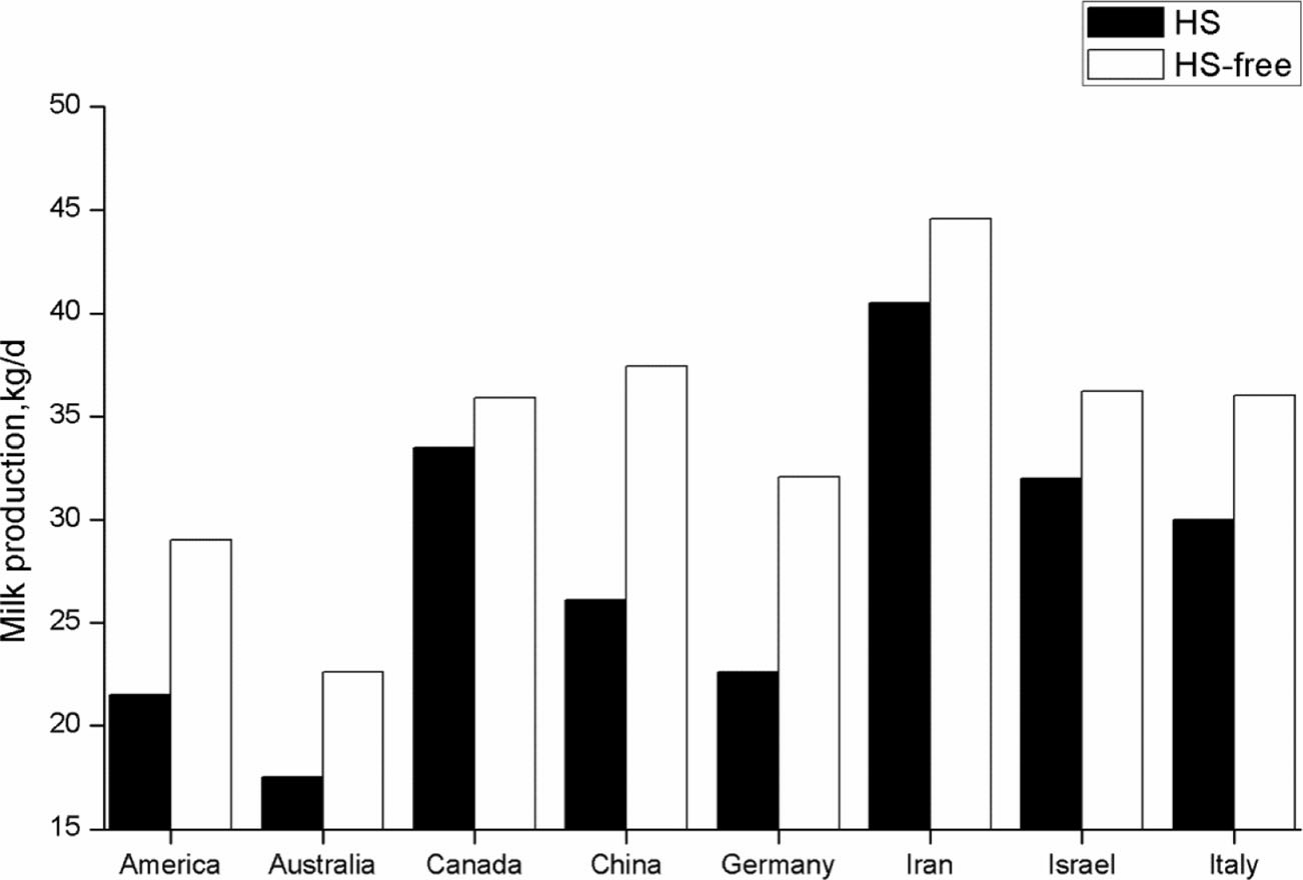
Summary of published studies showed the effect of HS and HS-free environment on milk production from different regions of the globe (Min et al. 2015; Rhoads et al. 2009; Cowley et al. 2015; Eslamizad et al. 2015; Ominski et al. 2002; Karimi et al. 2015; Flamenbaum and Galon 2010; Soriani et al. 2013). The data from Australia were converted as follows: 1 L/day = 1.0288 kg/day

HS negatively impacts on a variety of animal production parameters (Dunshea et al. 2013) and places a substantial financial burden on animal husbandry enterprises around the world (Bernabucci et al. 2010). In particular, dairy cows are extremely sensitive to a hot environment (Bernabucci et al. 2014). Despite advances in cooling systems and environmental management during the hotter seasons, HS continues to be a costly issue for the dairy industry (St-Pierre et al. 2003). As summarized in Fig. 2, dairy cows exposed to HS produce less milk compared to others kept under HS-free conditions in different regions of the globe (Min et al. 2015; Rhoads et al. 2009; Cowley et al. 2015; Eslamizad et al. 2015; Ominski et al. 2002; Karimi et al. 2015; Flamenbaum and Galon 2010; Soriani et al. 2013). A better understanding of the mechanisms whereby HS adversely affects dairy cows is necessary to increase the chances that suitable strategies will be developed to alleviate HS.

With the recent addition of more comprehensive research, much more is known about the effects of HS on dairy cows. Recent findings concern the effects of HS on lipid, carbohydrate, and protein metabolism, endocrine profiles and signaling proteins, and bovine mammary epithelial cell function. Metabolomics and proteomics approaches have revealed altered metabolite profiles and differentially expressed proteins that may play an important role in the HS response. This review will focus on each of these issues and describe a predictive HS metabolic model on whole organism, endocrine, and cellular levels, guided by the new insight afforded by “omics” technologies.

## The metabolic response to heat stress at whole organism, endocrine, and cellular levels

### Lipid, carbohydrate, and protein metabolism during heat stress

A prerequisite to understanding the metabolic changes that occur during HS is knowledge of metabolism under HS-free conditions. Compared with animals kept under HS-free conditions, HS results in decreases in plasma non-esterified fatty acid (NEFA) and glucose (Wheelock et al. 2010; Baumgard et al. 2011), whereas plasma lactate and urea nitrogen are increased (Tian et al. 2015; Shwartz et al. 2009).

Increased circulating plasma NEFA is typical of dairy cows on a lower plane of nutrition or in negative energy balance, when they provide a substantial source of energy and precursors for milk fat synthesis (Baumgard and Rhoads 2013). Despite the decrease in feed intake during HS (negative energy balance), it is counterintuitive that plasma NEFA decreased. The fact that HS cows decreased circulating plasma NEFA and lipolysis suggested that HS directly (not mediated by feed intake) impacts lipid and energetic metabolism. A series of studies demonstrated that plasma NEFA levels are typically reduced in animals with HS (Wheelock et al. 2010; Sano et al. 1983; Sanders et al. 2009; Pearce et al. 2011). Ronchi et al. (1999) hypothesized that the lower plasma NEFA concentrations in HS dairy cows were the result of elevated utilization rates of NEFA for energy production. The decrease of a NEFA response during HS may be a strategy to increase plasma insulin concentrations as excessive NEFA would cause pancreas β-cell apoptosis (Roche et al. 2000). The increase in plasma insulin concentrations during HS would mediate lipid metabolism, because insulin is a potent anti-lipolytic hormone that acts to maintain adipose lipogenesis (Wheelock et al. 2010). These observations are consistent with data from rats and cows that indicate that HS animals maintain a reduced rate of lipolysis (Torlińska et al. 1986; Bertoni 1998). It is also supported by HS pigs. The carcass data indicate that pigs have increased lipid retention and increased adipose tissue (lipid) when exposed to HS conditions (Baumgard and Rhoads 2013). Hence, the reduced rate of lipolysis in HS conditions is likely an evolutionary mechanism to reduce thermogenesis, because lipolysis (approximately 39.3 kJ/g) may generate more metabolic heat than that of carbohydrate (approximately 15.6 kJ/g) and protein (approximately 16.7 kJ/g) metabolism.

Streffer (1988) suggested that carbohydrate metabolism was altered during HS. The concentration of plasma glucose, a key precursor of milk components, is reduced during HS (Baumgard et al. 2011). As a result, HS dairy cows secrete approximately 200–400 g/day less milk lactose than cows kept under HS-free conditions (Wheelock et al. 2010). Instead, glucose is preferentially used for processes other than milk synthesis by HS animals (Streffer 1988; Baumgard and Rhoads 2013). On the other hand, plasma lactate concentrations are significantly higher in HS animals (Hall et al. 1980; Elsasser et al. 2009; Tian et al. 2015). The origin of this lactate is currently unknown, but it may be derived from the gastrointestinal tract or skeletal muscle (Baumgard and Rhoads 2013), where it would be the result of increased glycolysis to gain ATP immediately in response to HS.

In comparison with dairy cows kept under HS-free conditions, HS leads to increased plasma urea nitrogen levels (Shwartz et al. 2009). Plasma urea nitrogen can originate from two sources: decreased incorporation of rumen ammonia into microbial proteins, or from increased catabolism of amino acids as gluconeogenic substrates. In a recent experiment, no differences were observed in rumen ammonia concentration and microbial proteins between cows kept under HS-free and HS conditions (Cowley et al. 2015). Therefore, the increase in plasma urea nitrogen most likely results from catabolism of amino acids. It was postulated that amino acids, which would be a key precursor of milk protein synthesis, were also being scavenged for gluconeogenic purposes, reducing the precursor pool for the synthesis of milk proteins (Cowley et al. 2015). These consequences suggest that the metabolism of protein (amino acids) and carbohydrate (glucose) is used in preference to lipid (NEFA) in HS conditions in order to obtain the carbon backbone for gluconeogenesis and meet the energy needs of dairy cows, respectively.

### Adaptive changes in endocrine and signaling proteins in response to heat stress

Metabolic adaptations to HS in dairy cows are likely driven by changes in the endocrine system and associated signaling proteins, with the goal of maintaining homeostasis in the face of HS challenge. Adaptation to HS involves an altered endocrine status that ultimately affects target tissue responsiveness to HS stimuli (Bernabucci et al. 2010). Meanwhile, HS response for acclimation include activation of heat shock transcription factor (HSF), increased expression of heat shock proteins (HSPs), and adenosine 5′-monophosphate (AMP)-activated protein kinase (AMPK) (Collier et al. 2008; Min et al. 2015). Additional changes in endocrine and signaling proteins recorded during HS in dairy cows are listed in Table 1.

**Table 1:** Partial list of endocrine and signaling proteins changes during HS dairy cows

Tissue/signaling proteins	Response	Reference
Pancreas	Increased insulin secretion	Wheelock et al. (2010) and O’Brien et al. (2010)
Adipose tissue	Increased leptin secretion	Bernabucci et al. (2006)
	Increased adiponectin secretion	Min et al. (2015)
AMPK	Activated AMPK	Min et al. (2015)
HSF	Activated HSF	Page et al. (2006) and Trinklein et al. (2004)
HSPs	Activated HSPs	Collier et al. (2008) and Gaughan et al. (2013)

Insulin is the primary anabolic endocrine signal, and it plays a critical role in lipid, carbohydrate, and protein metabolism (Baumgard et al. 2016). Despite HS significantly reducing feed intake, which would be expected to result in reduced insulin secretion, HS actually stimulates insulin secretion (O’Brien et al. 2010; Wheelock et al. 2010). Rhoads et al. (2013) showed that proper insulin signaling and action is necessary to mount an effective response to HS and minimize heat-induced damage. Diabetic (hypoinsulinemia) rats show an increased mortality rate when exposed to HS, while their survival time is increased after exogenous insulin treatment (Niu et al. 2003). The reasons for the hyperinsulinemia during HS are not clearly understood, but may include the key role of insulin in activating and up-regulating HSPs (Li et al. 2006), which are important mediators of insulin action and sensitivity (Geiger and Gupte 2011). Consequently, the increased insulin may be an essential part of the adaptive mechanism and play a critical role during HS (Baumgard and Rhoads 2013).

It is clear that adipose tissue plays a critical role in the regulation of lipid and carbohydrate metabolism (Bernabucci et al. 2009a). Two adipokines (leptin and adiponectin), which are secreted by adipose tissue, are metabolically relevant in coordinating energy homeostasis (Ailhaud 2006). Chronic HS up-regulates leptin and adiponectin secretion and improves leptin, adiponectin, and insulin sensitivity in mice (Morera et al. 2012). Bernabucci et al. (2006) also showed that HS increases leptin secretion in periparturient dairy cows, while Min et al. (2015) showed that HS increases the concentrations of adiponectin in dairy cows. The molecular mechanisms whereby HS induces changes in the expression of adipokines remain unclear. Park et al. (2005) suggested that HS causes changes in the fluidity of membrane lipids, which might induce the heat shock response, characterized by increased expression of HSPs, which could directly stimulate adipokine expression. Moreover, plasma leptin and adiponectin levels are positively correlated with insulin sensitivity (Rabe et al. 2008), and adiponectin directly sensitizes tissues to insulin and stimulates insulin secretion by the pancreas (Kadowaki et al. 2006). The up-regulation of leptin and adiponectin expression is probably one of the mechanisms involved in the adaptive thermoregulatory processes that might be what is responsible to better resist the damaging effects of HS (Houseknecht et al. 1998; Hoyda et al. 2011).

We showed previously that activity of serum AMPK is increased in HS dairy cows (Min et al. 2015). AMPK is a signaling protein that plays a key role in the regulation of energy balance (Carling 2005). It is involved in many types of stress response and thus can be regarded as an indicator of stress. Although the mechanism for AMPK release into serum is still ill-defined, analysis of serum AMPK may assist with the diagnosis of metabolic diseases (Malvoisin et al. 2009). Indeed, Frederich et al. (2009) concluded that AMPK is an earlier indicator of HS in rock crabs. Furthermore, AMPK activity is strongly associated with adipokines. Leptin and adiponectin both activate AMPK and thereby modify usage of metabolic pathways (Andersson et al. 2004; Klimcakova et al. 2006). Yamauchi et al. (2002) demonstrated that adiponectin activates AMPK, thereby directly regulating glucose metabolism and insulin sensitivity in vitro and in vivo. Consequently, the increase in adiponectin in dairy cows with HS would be expected to increase AMPK activation, a mechanism that may be involved in metabolic adaptation during HS.

The HS response is fully integrated with the physiological stress response, and dairy cows have a protective mechanism against thermal stress that involves activation of HSF and increased expression of HSPs (Collier et al. 2008). HSF is a transcription factor that has been demonstrated as important first responders during HS (Page et al. 2006; Trinklein et al. 2004). Subsequently, HSF increases the expression of HSPs, which are molecular chaperones that promote the refolding of unfolded or misfolded proteins (Li et al. 2011). Thus, overexpression of HSPs protects against hyperthermia and maintains homeostasis (Lee et al. 2006). In addition to their protective role, HSPs may enhance insulin function. Rhoads et al. (2013) suggested that the activation of the insulin-HSP axis may improve animal health and productivity during HS. Among all the HSPs, expression levels of HSP70 are most closely indicative of the magnitude of HS (Tanaka et al. 1988), and Gaughan et al. (2013) suggested that the plasma concentration of HSP70 is a reliable indicator of HS. This contention was supported by Min et al. (2015), who evaluated differences in expression of signaling proteins among cows with moderate, mild, and no HS, showing that HSP70 levels are more regulated in HS than those of other HSPs. Similar results were also obtained in bovine mammary epithelial cells, in which HSP70 is extremely sensitive to HS and is mainly responsible for protection of mammary cells from HS (Hu et al. 2016b). Hence, we recommend HSP70 as a biomarker to monitor HS.

### The effects of heat stress on bovine mammary epithelial cells

HS results in a decrease in milk production. The mammary secretory function in dairy cows is dependent on the number of mammary epithelial cells and their secretory activity (Miller et al. 2006). Mammary epithelial cells can directly be affected by hyperthermia. On a cellular level, bovine mammary epithelial cells exhibit morphological changes and reduced cellular growth when kept at 42 °C, alongside reduced expression of genes involved in protein synthesis and cellular metabolism (Collier et al. 2006). Recently, Hu et al. (2016a) indicated that HS induces cell apoptosis and disturbs normal biological activity. These direct detrimental effects on the mammary gland would be expected to result in decreased milk production. In addition to reducing milk synthesis during HS, milk characteristics have also been shown to be affected by HS. Specifically, HS reduces milk protein concentration, casein number, and casein concentration (Cowley et al. 2015). Casein fractions (α-casein, β-casein, and κ-casein), with the exception of γ-casein, showed the lowest values in HS (Bernabucci et al. 2015). These phenomena have also been shown clearly in vitro; HS down-regulated the expression of major milk protein genes (β-casein and butyrophilin) and decreased the synthesis of total caseins in bovine mammary epithelial cells (Hu et al. 2016b). Indeed, the direct effects on the mammary epithelial cells would result in the decrease in milk protein, but there was no significant difference for other milk compositions in our previous study (Cheng et al. 2014). Presumably, HS negatively affects the synthesis of milk protein in mammary epithelial cells, but the direct action might contribute little to the decrease in other milk compositions and milk yield. The decrease in milk yield in HS conditions may mainly contribute to the metabolic response at extra-mammary as described above.

## “Omics” technologies for elucidating the effects of heat stress in dairy cows

“Omics” is a term that collectively refers to recently developed high-throughput technologies that include genomics, transcriptomics, proteomics, and metabolomics. These state-of-the-art technologies can be used to evaluate metabolism in a whole organism, tissue, or cell at a molecular level. Application of omics approaches to livestock has great potential for understanding what happened and how to improve efficiency of animal production. In recent years, many researchers have investigated the gene expression, proteomics, and metabolomics of HS in dairy cows. These findings have expanded current knowledge to understanding the molecular mechanisms underpinning HS in dairy cows and offer a new perspective for future research.

### Integrated metabolomics study of the plasma and milk in HS dairy cows

**Fig. 3:**
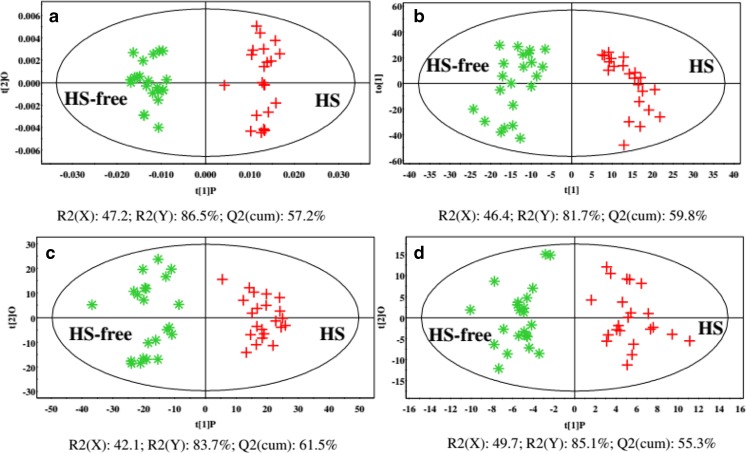
The discrimination of plasma metabolic profiles between HS and HS-free dairy cows using multivariate analysis by metabolomics analysis (Tian et al. 2015). **a** OPLS-DA plots of NMR data. **b** OPLS-DA plots of LC–(+)ESI/MS data for the plasma metabolomes. **c** OPLS-DA plots of LC–(−)ESI/MS data for the plasma metabolomes. **d** OPLS-DA plots of LC–(+)ESI/MS data for the plasma lipidomes

Metabolomics represents a powerful platform for the identification of metabolites as biomarkers associated with physiological alterations resulting from environmental influences. Integrated ^1^H nuclear magnetic resonance (NMR) and liquid chromatography-mass spectrometry techniques were employed to comprehensively investigate the discrimination of plasma metabolic profiles between HS and HS-free dairy cows (Tian et al. 2015). The orthogonal partial least squares discriminate analysis (OPLS-DA) plots show a clear separation in the metabolic profile between HS and HS-free dairy cows without any overlap (Fig. 3). Hence, it is obviously that HS induced metabolic alterations in dairy cows. Overall, 41 metabolites were identified as metabolic differences during HS, and all of these potentially biomarkers were involved in pathways of carbohydrate, amino acids, lipid, and gut microbiome-derived metabolism. This study clearly showed that HS results in the decrease in plasma glucose and an increase in plasma lactate and lactate dehydrogenase activity in dairy cows. The increase in the metabolite and enzyme activity of glycolysis may suggest that there is an increase in glycolysis in HS conditions. Combined with the increase in respiration rate in HS dairy cows, we hypothesized that enhanced anaerobic cell respiration and glycolysis may be an adaptive mechanism to generate ATP immediately in response to HS. The concentrations of proline, glycine, threonine, isoleucine, and arginine were increased in HS dairy cows. These amino acids may provide precursors for gluconeogenesis and meet the energy needs of the dairy cows during HS, as discussed above. This study also showed that HS results in increased concentrations of trimethylamine, trimethylamine oxide, and isobutyrate which are associated with gut microbiome-derived metabolism. The precise role of gut microbiome-derived metabolism alterations in HS dairy cows is still unknown. To date, the effects of heat stress on microbiome in dairy cows have not yet been clearly elucidated, especially on rumen microbiome. Uyeno et al. (2010) evaluated the effects of HS on the rumen microbial composition of Holstein heifers. The results showed that HS significantly increased the abundance of *Lactococcus* in heifers. As already mentioned, HS animals have a “leaky gut” (Mani et al. 2012). The intestinal barrier function compromised in HS conditions may associate with the gut microbiome response, which showed the increase in lactate-producing bacteria. Furthermore, the faster growth of lactate-producing bacteria might be a reason for the increase in plasma lactate observed in HS.

Subsequently, an integrated metabolomics study was performed on the milk of HS dairy cows (Tian et al. 2016). The metabolites that were modified by HS in milk were also involved in carbohydrate, amino acid, lipid, and gut microbiome-derived metabolism. Comparing the difference metabolites in milk with previously identified biomarkers in plasma during HS, significant correlations between the levels of lactate, pyruvate, creatine, acetone, β-hydroxybutyrate, trimethylamine, oleic acid, linoleic acid, lysophosphatidylcholine 16:0, and phosphatidylcholine 42:2 were found, indicating that the blood-milk barrier may become more permeable, and these 10 biomarkers in milk may represent the metabolomics alterations in blood during HS. It is noteworthy that HS results in the increase in lactate in both plasma and milk, further reflecting enhanced anaerobic glycolysis in HS dairy cows.

### Proteomics analysis further characterizes the metabolic response in HS dairy cows

The metabolomics experiment discussed above analyzed the discrimination of plasma metabolic profiles of HS versus HS-free dairy cows. Meanwhile, the plasma proteomics profiles of HS and HS-free dairy cows were undertaken using isobaric tags for relative and absolute quantification. Results showed that many factors in the complement system (including complement components C1, C3, C5, C6, C7, C8, and C9, and complement factor B and factor H) are down-regulated in blood by HS (Min et al. 2016a). The complement system is a part of the immune system that plays a fundamental role in innate immunity in addition to enhancing adaptive immune responses and is the primary line of defense against infection (Carroll 2008). A series of studies have indicated that HS reduces immune function in dairy cows. HS impairs innate and acquired immune status in the transition period of dairy cows, indicated by defects in neutrophil function and immunoglobulin secretion (do Amaral et al. 2011). Furthermore, HS of the dam during the dry period compromises the passive immunity of offspring from birth through weaning, suggesting that the immune function can also be compromised in calves (Tao et al. 2012). However, the precise mechanisms underlying impaired immune function in lactating dairy cows during HS remain undefined, particularly with regard to complement system profiles. HS decreases the abundance of components of the plasma complementary system, suggesting that HS results in impaired immune function in lactating dairy cows and expands the current knowledge on the effects of HS on immune function of dairy cows.

Furthermore, the proteomics results were highly consistent with the results of the previous metabolomics study (Table 2). The plasma concentrations of phosphatidylcholine (PC) (16:0/14:0), PC (14:1/18:3), PC (12:0/22:2), PC (15:1/18:2), PC (20:2/12:0), and PC (18:1/18:3) are lower in HS relative to HS-free cows (Tian et al. 2015), and plasma phosphatidylcholine-sterol acyltransferase, apolipoprotein B-100, apolipoprotein A-I, and apolipoprotein A-II are down-regulated in HS cows (Min et al. 2016a). The down-regulation of these apolipoproteins and the enzyme phosphatidylcholine-sterol acyltransferase, which belonged to lipoproteins, suggests that lipid transport is blunted in HS dairy cows. This result agrees with the rat data (Torlińska et al. 1986) and the metabolic responses in HS dairy cows discussed above. In addition, Basiricò et al. (2009) indicated that HS strongly down-regulated apolipoprotein B-100 gene and protein expression in transition cows, and the incidence of fatty liver is higher. Coupled with the fact that HS cows showed a blunted adipose tissue mobilization (Wheelock et al. 2010), it strongly suggests that the increase in fatty liver without increased adipose mobilization and the reduced rate of lipolysis would happen in HS cows. Aminoacylase-1 was up-regulated in the plasma of HS dairy cows (Min et al. 2016a). This enzyme catalyzes the hydrolysis of *N*-acetylated peptides and is involved in the final release of free amino acids (Perrier et al. 2005). The up-regulation of aminoacylase-1 during HS might catalyze more free amino acids into the plasma, which is in agreement with the altered amino acid metabolic profiles identified in the metabolomics study (Tian et al. 2015). The results presented here further suggest that HS may cause nitrogen repartitioning in dairy cows. Thus, through integrative analyses of plasma proteomics and metabolomics data and the metabolic response discussed above, we have provided strong evidence of reduced lipolysis, increased glycolysis, and catabolism of amino acids in dairy cows exposed to HS.

**Table 2:** Integrative analyses of plasma proteomics and metabolomics results to show the lipid, carbohydrate, and protein metabolism in HS dairy cows

Item	Proteomics (Min et al. 2016a)	Metabolomics (Tian et al. 2015)	Metabolic response
Lipid metabolism	Phosphatidylcholine-sterol acyltransferase, apolipoprotein B-100, apolipoprotein A-I, and apolipoprotein A-II **↓**	Phosphatidylcholine (PC) (16:0/14:0), PC (14:1/18:3), PC (12:0/22:2), PC (15:1/18:2), PC (20:2/12:0), and PC (18:1/18:3) **↓**	Lipolysis **↓**
Carbohydrate metabolism	Lactate dehydrogenase **↑**	Lactate **↑**	Glycolysis **↑**
Protein metabolism	Aminoacylase-1 **↑**	Proline, glycine, threonine, isoleucine, and arginine **↑**	Catabolism of amino acids **↑**

In contrast to short-term HS, long-term HS occurs more commonly in the natural world. Bernabucci et al. (2009b) compared the effects of short term- and long-term HS, finding that respiratory rate and rectal temperature increase immediately in short-term HS but gradually return to normal values after long-term HS. Indeed, the physiological responses to long-term HS in dairy cows are few. The plasma proteomics profiles and western blot analysis showed that long-term HS significantly decreases the expression of transthyretin in dairy cows (Min et al. 2016b), and the synthesis of transthyretin is known to be reduced by inflammation (Ceciliani et al. 2012). Furthermore, long-term HS significantly increases plasma tumor necrosis factor-α and interleukin-6, which are pro-inflammatory factors. Hence, it is reasonable to conclude that long-term HS induces an inflammatory response in dairy cows. Further research is therefore required to corroborate these findings and investigate whether alleviation of inflammation may improve the deleterious effects of HS.

## Conclusions

Characterization of lipid, carbohydrate, and protein metabolism in heat-stress dairy cows showed that dairy cows would maintain a reduced lipolysis, enhanced glycolysis to maintain energy homeostasis, and increased use of amino acids for gluconeogenesis. These results were supported by metabolomics and proteomics analysis of body fluids. The increased of insulin, adipokines, AMPK, HSF, and HSPs also play a critical role in resisting the damaging effects of HS. Together, these findings demonstrate that metabolism and the endocrine system are adjusted to minimize the detrimental effects of HS in dairy cows. However, omics analysis revealed that HS still induces a decrease in complement components, suggesting that HS dairy cows have impaired immune function. Moreover, long-term HS induces the inflammation in dairy cows. These findings offer a novel perspective in the search for effective approaches to ameliorate HS in dairy cows.
